# A Target Detection Algorithm for Remote Sensing Images Based on Deep Learning

**DOI:** 10.1155/2021/3474921

**Published:** 2021-12-18

**Authors:** Yi Lv, Zhengbo Yin, Zhezhou Yu

**Affiliations:** ^1^College of Computer Science and Technology, Jilin University, Changchun, Jilin 130000, China; ^2^College of Computer Science and Technology, Changchun Normal University, Changchun, Jilin 130000, China; ^3^College of Innovation and Entrepreneurship, Changchun University of Chinese Medicine, Changchun, Jilin 130000, China

## Abstract

In order to improve the accuracy of remote sensing image target detection, this paper proposes a remote sensing image target detection algorithm DFS based on deep learning. Firstly, dimension clustering module, loss function, and sliding window segmentation detection are designed. The data set used in the experiment comes from GoogleEarth, and there are 6 types of objects: airplanes, boats, warehouses, large ships, bridges, and ports. Training set, verification set, and test set contain 73490 images, 22722 images, and 2138 images, respectively. It is assumed that the number of detected positive samples and negative samples is *A* and *B*, respectively, and the number of undetected positive samples and negative samples is *C* and *D*, respectively. The experimental results show that the precision-recall curve of DFS for six types of targets shows that DFS has the best detection effect for bridges and the worst detection effect for boats. The main reason is that the size of the bridge is relatively large, and it is clearly distinguished from the background in the image, so the detection difficulty is low. However, the target of the boat is very small, and it is easy to be mixed with the background, so it is difficult to detect. The MAP of DFS is improved by 12.82%, the detection accuracy is improved by 13%, and the recall rate is slightly decreased by 1% compared with YOLOv2. According to the number of detection targets, the number of false positives (FPs) of DFS is much less than that of YOLOv2. The false positive rate is greatly reduced. In addition, the average IOU of DFS is 11.84% higher than that of YOLOv2. For small target detection efficiency and large remote sensing image detection, the DFS algorithm has obvious advantages.

## 1. Introduction

Target detection is an important part of image processing, especially for remote sensing images. Target detection in remote sensing images plays an important role in both military and civil fields [1]. In the civil field, remote sensing technology is widely used in resource survey, urban planning, crop yield estimation, and so on. In the military field, remote sensing technology has become an important means of military investigation and early warning in modern army. Remote sensing technology has many advantages [2]. With the deepening of machine learning, especially deep learning, deep learning has become an important means of image target detection. A large number of satellite remote sensing images provide sufficient samples for deep learning target detection. A large number of results show that the deep learning algorithm has the ability to process a large amount of information quickly and accurately when there are sufficient samples. Therefore, through the remote sensing image target detection system based on deep learning and automatic extraction means, a large number of satellite remote sensing images can be processed quickly and accurately and the effective information can be mined, which can avoid the shortcomings of traditional target detection algorithms and improve the performance of target detection. As a typical problem in the field of computer graphics and computer vision, remote sensing target detection has been studied by many foreign scholars in recent ten years, and some good research results have been yielded [3]. Target detection algorithms can be roughly divided into the period based on traditional manual features (before 2013) and the period based on deep learning (2013∼present). In terms of technical development, the development of target detection has experienced “bounding box regression,” “the rise of deep neural network,” “multireference,” “mining and focusing difficult samples” and “multiscale and multiport detection.” Traditional target detection algorithms are mostly based on manual features [4]. Due to the lack of effective image feature expression methods, we have to try our best to design more diversified detection algorithms to make up for the defect of manual feature expression ability [5]. At the same time, due to the lack of computing resources, we have to find more sophisticated computing methods to accelerate the model. Feature extraction and classifier classification are the key steps of traditional target detection algorithms. Various modifications and improvements of traditional target detection algorithms are also focused on these two aspects [6]. Theoretically, because the target is affected by different lighting conditions, shooting angles, and shooting distances, the target features are inconsistent. At the same time, the nonrigid changes of target objects will also cause the difference of target features and the target detection algorithm needs to find these targets, which is challenging to some extent. In recent years, the development of deep learning has improved the accuracy of target detection to a certain extent, but there are still the following problems: the detection accuracy and efficiency of large-format and high-resolution images are low. In the complex scene with a small sample set, the performance of target detection is low and the factors that cause the low accuracy of target detection are mainly manifested in the following three aspects: large-format and high-resolution images bring more and clearer information but also increase the difficulty of target detection. There are many small target objects in large-format high-resolution images, and the detection accuracy is low. In addition, high-resolution images will introduce more calculations, which reduce the real-time performance of target detection [7]. Shooting images in different environments leads to problems such as diverse target postures and complex target background, which also greatly increases the difficulty of target detection. In the application fields of robot service, remote sensing image detection, and medical image, the number of training samples is small and the performance of target detection under the small sample set is poor in accuracy, which restricts the application range of target detection. These problems need to be further considered and solved in the target detection algorithm. According to this research question, Ilyes believed that the method of using regression to detect targets became a new research hotspot. The classification problem was transformed into regression problem, which simplified the whole target detection process and greatly improves the running speed of the whole system. YOLO combined target determination and target recognition into one, which greatly accelerated the detection speed. Because the whole map information was used for network prediction, the false alarm rate was low. However, the algorithm also had the problems of inaccurate positioning and low detection accuracy. The SSD model integrated regression thought and multiwindow mechanism and used multiscale regional features for regression, which not only ensured the detection speed but also ensured the detection accuracy [8]. Kalyzhner et al. proposed a deformable part model (DPM) based on classical manual features. DPM splatted the detection problem of the whole target in the traditional target detection algorithm into the detection problem of each part of the model and then aggregated the detection results of each part to obtain the final detection result, which was a process of “from whole to part, and then from part to whole.” The whole DPM detector consisting of a base filter and a series of component filters was optimized by the strategy of weak supervised learning [9]. Zhang et al. improved the model and further transformed it into the optimization problem of hidden variable structure SVM, which was solved by combining difficult sample mining and stochastic gradient optimization strategy. The linear SVM classifier in DPM is “compiled” into a series of cascaded decision pile classifiers for model acceleration. The DPM algorithm used bounding box regression and context information integration to further improve the detection accuracy. The bounding box corresponding to the detected base filter and component filter was integrated, the final accurate bounding box coordinates were obtained by linear least square regression, and the detection results were readjusted by using global information [10]. On the basis of current research, this paper proposes a remote sensing image target detection algorithm DFS based on deep learning. Firstly, dimension clustering module, loss function, and sliding window segmentation detection are designed. The data set used in the experiment comes from GoogleEarth, and there are 6 types of objects: airplanes, boats, warehouses, large ships, bridges, and ports. Training set, verification set, and test set contain 73490 images, 22722 images, and 2138 images, respectively. It is assumed that the number of detected positive samples and negative samples is *A* and *B*, respectively, and the number of undetected positive samples and negative samples is *C* and *D,* respectively. For small target detection efficiency and large remote sensing image detection, DFS algorithm has obvious advantages.

## 2. Methods

### 2.1. DFS Target Detection Algorithm

It is mainly composed of neural network, dimension clustering, image segmentation, and other modules. The neural network module is responsible for target location and classification of the input image. The dimension clustering module is responsible for designing and selecting candidate frames. The image segmentation module is responsible for segmenting the original image, and the loss function module is located in the neural network module which is used to optimize the loss function. DFS designs a new dimension clustering, loss function, and sliding window segmentation detection mechanism. Among them, the dimension clustering mechanism makes full use of the prior information of the training set and designs a new prior frame mechanism, which effectively improves the positioning accuracy. Aiming at the problem that the remote sensing images of small targets are difficult to detect, a new Focalloss function is designed. Aiming at the problem of low accuracy of large image detection, a sliding window segmentation detection mechanism is designed.

### 2.2. Dimension Clustering Module

It is an unsupervised learning process of searching clusters, and cluster analysis is usually carried out as preparation work in data mining. *K*-means is a commonly used clustering algorithm, and the evaluation standard of its similarity is the distance between data. The algorithm thinks that the closer the distance between two target points, the greater their similarity. The prior box used in the current single-stage target detection algorithm is designed for medium and large targets, which has a big gap with the detection requirements of small and dense targets in remote sensing data sets. Therefore, it is necessary to recalculate and select the appropriate prior box to help model learning. DFS algorithm adds the dimension clustering module to calculate the prior box for remote sensing image target detection. The function of the dimension clustering module in DFS algorithm is to extract the labeled frames in the training set and cluster them [11]. The width and height (*w*, *h*) of each marker box were taken as sample data, and the width and height (*C*_*w*_*C*_*h*_) of AnchorBoxes were defined as clustering centers. The goal of clustering is that the size and proportion of the obtained prior frame are as close as possible to those of the labeled frame in the training set, that is, IOU is as large as possible, so the distance in the clustering algorithm is defined as shown in

(1) $$\begin{array}{c} d\left(\text{box},\text{centroid}\right)=1-\text{IOU}\left(\text{box},\text{centroid}\right). \end{array}$$

Here, IOU(box, centroid) refers to the IOU of the prior box and the marker box centroid generated by clustering, that is, the ratio of intersection and union between the predicted frame and the real frame. The larger the value, the smaller the distance, that is, the more likely it is to cluster into the same cluster. Detailed process description is as follows:

1. Giving a training set *d*, the width and height (*w*, *h*) of the marker box of each target are obtained as sample points
2. Randomly specify the width and height (*C*_*w*_*C*_*h*_) of *k* prior boxes as the clustering centers of *K* subsets
3. According to the distance between the sample points and the *k* cluster centers, each sample point is classified into the subset where the nearest cluster center is located
4. Recalculate cluster centers for these *k* subsets
5. Repeat the operation in step (3) according to the new clustering center
6. Repeat steps (4) and (5) until the results converge

The above algorithm is sensitive to initialization. Because random initialization is adopted, the initial clustering centers may be close, which may affect the division of subsets. In view of this shortcoming, this paper improves the initialization method, so that the distance between cluster centers is as far as possible during initialization. The specific process of selecting the initial cluster center is as follows:

1. Randomly select the width and height (*w*, *h*) of a marker box from the training set *d*, and set it as the first clustering center
2. For every sample point *x* in the training set, calculate the distance d(*X*) between it and the nearest cluster center (referring to the selected cluster center)
3. The following principles are adopted to select a new cluster center: the larger the point d(*X*), the higher the probability of being selected as a cluster center
4. Repeat (2) and (3) until all *k* cluster centers are selected. After *k* initial clustering centers are obtained by the above method, they are used as the input of dimensional clustering algorithm and the optimal prior box is generated.

### 2.3. Loss Function Design

Loss function refers to the function used to calculate the difference between the real value and the predicted value. The essence of machine learning is to train the model through training samples and get reasonable weights. The evaluation of the training process depends on the loss function value, and the weight in the training process is adjusted according to it [12]. In each image, when calculating the loss function, the boundary box can be divided into positive and negative samples. Generally, the proportion of objects in images is much smaller than that of backgrounds, so negative samples are the main ones in the two types of samples. This leads to too many negative samples, which is not conducive to the convergence of the target, and most of the negative samples are not in the transition area between the foreground and background, so the classification is very clear. The information that makes it difficult to distinguish the sample from the positive sample is concealed [13].

This loss function reduces the accuracy of the single-stage detection method, especially in remote sensing images. Therefore, DFS introduces Focalloss function to replace the original loss function. Focalloss is improved on the basis of the standard cross entropy loss function, which is a special logarithmic loss function and is often used in multicategory regression tasks. Its square error loss function are the most commonly used loss functions in classification and detection tasks, and their update gradient is larger, which can accelerate the convergence of neural networks and shorten the training time. The formula of cross entropy loss function is shown as follows:

(2) $$\begin{array}{c} \text{CE}=-\frac{1}{n}\sum_{x}\left[y\;\ln\hat{y}+\left(1-y\right)\ln\left(1-\hat{y}\right)\right]. \end{array}$$

Here, *y* represents the predicted value, $\hat{y}$ represents the true value, *x* represents the sample value, and *n* represents the total number of samples.

Taking the two-classification problem as an example, the standard cross entropy loss function is defined as shown in

(3) $$\begin{array}{c} \text{CE}\left(p,y\right)=\left\{\begin{array}{cc} -\log\left(p\right), & l=1,\\ -\log\left(1-p\right), & l=-1, \end{array}\right) \end{array}$$

where *l* represents the true label of the sample, 1 represents the positive sample, and −1 represents the negative sample. *p* represents the probability of the target category, and its value is (0,1). Equation (3) shows that when the sample is a positive sample, the larger the sample is, the smaller the loss function will be and the better the network detection effect will be. When the sample is negative, the smaller the *p*, the smaller the loss function and the better the network detection effect. For ease of presentation, *p*_*t*_ is defined as the closeness between the predicted value of the network and the true value, as shown in

(4) $$\begin{array}{c} p_{t}\left\{\begin{array}{cc} p, & l=1,\\ 1-p, & l=-1. \end{array}\right) \end{array}$$

Then, the cross entropy loss function can be expressed as shown in

(5) $$\begin{array}{c} \text{CE}\left(p_{t}\right)=-\log\left(p_{t}\right). \end{array}$$

In order to solve the problem of category imbalance, it is necessary to adjust the contribution value of different categories to the loss function, that is, to add a control weight to the loss function *a*_*t*_. Considering that the easier the sample is to distinguish, the higher the probability of classification will be (*p*_*t*_) [14]. In order to restrain the learning of easily distinguishable samples and strengthen the learning of indistinguishable samples, a regulation factor (1 − *p*_*t*_)^*γ*^ is added to the loss function. When *p*_*t*_ is larger, the loss function is multiplied by a smaller factor. When *p*_*t*_ is smaller, the loss function is multiplied by a larger factor. *γ* can play a role in regulating the degree of inhibition (or enhancement). The final Focalloss function is defined as follows:

(6) $$\begin{array}{c} \text{FL}\left(p_{t}\right)=-a_{t}{\left(1-p_{t}\right)}^{\gamma }\log\left(p_{t}\right). \end{array}$$

Here, *p*_*t*_ is the classification probability of different categories. *γ* is a value greater than 0, *a*_*t*_ ∈ [0,1]. *γ* and *a*_*t*_ are the fixed value and do not participate in training. It can be seen from formula (6) that whether it is foreground class or background class, the greater the *p*_*t*_, the smaller the (1 − *p*_*t*_)^*γ*^. It means that the sample belongs to “simple sample,” and the learner has been able to judge the true category of the sample well, so it is not necessary to give it a higher weight to learn it. Focalloss makes its influence smaller in the whole training process by reducing the loss value produced by such samples [15]. In addition, for the binary classification problem, *a*_*t*_ is used to adjust the ratio of positive and negative samples and used when the category is positive sample. *a*_*t*_ is used when the category is a negative sample.

### 2.4. Sliding Window Segmentation Detection

Most remote sensing images are stored in tif format, which has a large amount of information and high resolution. However, the single-stage target detection algorithm will normalize the size of the input image. Therefore, if remote sensing images are directly taken as input, many small targets will be lost. In this section, a block detection method is designed to solve this problem. It adds two steps in the detection stage:

1. For any size image, use sliding window to segment, and the slice size is the normalized image size (416*∗*416).
2. Input the slices into the original network for detection. After the detection, the sections were spliced and reassembled according to the original position. This segmentation detection method reduces the information loss caused by normalization, makes the size selection of input images more flexible, and greatly improves the algorithm performance.

### 2.5. Experimental Preparation

The data set used in the experiment comes from GoogleEarth, and there are 6 types of objects: airplanes, boats, warehouses, large ships, bridges, and ports. Training set, verification set, and test set contain 73490 images, 22722 images, and 2138 images respectively.

Assume that the number of detected positive samples and negative samples is *A* and *B*, respectively, and the number of undetected positive samples and negative samples is *C* and *D*, respectively. The experimental indexes are shown in Table 1. MAP refers to the area under the curve in the precision-recall graph, which indicates the average accuracy of the detection results and is also the most commonly used performance index [16].

## 3. Results and Analysis

The model is trained by using the set parameters, and the loss function tends to be stable after 10,000 rounds of iteration. The result is taken as the final training weight model, which is tested by the test set [17].

**Figure 1:**
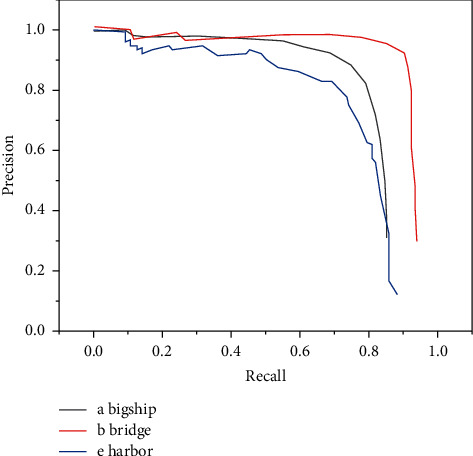
Precision-recall rate curves of large ships, bridges, and ports.

**Figure 2:**
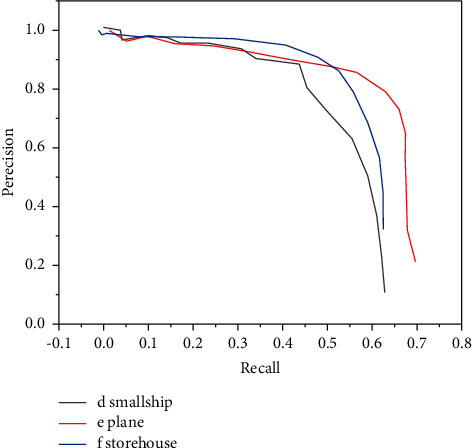
Precision-recall rate curves of boats, airplanes, and warehouses.

The precision-recall curves of six types of objects in the verification set are shown in Figures 1 and 2.

It can be seen from the precision-recall curve of DFS for six types of target detection that DFS has the best detection effect for bridges and the worst detection effect for boats. The main reason is that the size of the bridge is relatively large and it is clearly distinguished from the background in the image, so the detection difficulty is low. However, the target of the boat is very small and it is easy to get mixed with the background, so it is difficult to detect [18]. Comparing the detection effects of YOLOv2 algorithm and DFS algorithm on the test set, the results are shown in Table 2.

The experimental results show that the MAP of DFS is 12.82% higher than that of YOLOv2, the detection accuracy is 13% higher than that of YOLOv2, and the recall rate is slightly reduced by 1% [19]. According to the number of detection targets, the number of false positives (FPs) of DFS is greatly reduced compared with that of YOLOv2, and the false positive rate for positive cases is greatly reduced. In addition, the average IOU of DFS is 11.84% higher than that of YOLOv2, which is due to the adoption of the dimensional clustering method in DFS. Nine more accurate prior frames are selected, while YOLOv2 has only five prior frames and its targeting effect is poor. The better number and quality of prior frames make the average IOU of DFS significantly improved. For the detection of large remote sensing images, DFS algorithm has obvious advantages [20].

## 4. Conclusion

Aiming at the inaccuracy of target detection in remote sensing images, a new target detection algorithm DFS is proposed, which is mainly composed of neural network, dimensional clustering, image segmentation, and other modules. Dimension clustering module, loss function, and sliding window segmentation detection are designed. Nine more accurate prior frames are selected, while YOLOv2 has only five prior frames and its targeting effect is poor. Better number and quality of prior frames make the average IOU of DFS significantly improved. For the detection of large remote sensing images, DFS algorithm has obvious advantages.

## Figures and Tables

**Table 1:** Experimental indicators and their calculation methods.

Index	Calculation method
Accuracy rate	*p*=(*A*/(*A*+*B*))
Recall rate	*R*=(*A*/(*A*+*C*))
*f*1 score, *f* score, and *f* measure	*F*_1_ − score=(2*PR*/(*P*+*R*))
Average precision	*mAP*=∫_0_^1^*P*(*R*)d*R*

**Table 2:** Performance comparison between DFS and YOLOv2.

Algorithm	Accuracy rate	Recall rate	Average IOU	mAP
YOLOv2	72	55	55.70	50.17
DFS	85	54	67.54	62.99

## Data Availability

The data used to support the findings of this study are available from the corresponding author upon request.
